# Association between low water intake and lung function impairment in elderly adults: a cross-sectional study

**DOI:** 10.5588/pha.25.0038

**Published:** 2026-03-06

**Authors:** P.-C. Wang, Y.-F. Wu, M.-S. Lin, Y.-C. Tu, S.-Y. Huang, J.-H. Lung, C.-S. Shi, M.-Y. Chen

**Affiliations:** 1 Department of Cardiology, Chang Gung Memorial Hospital, Chiayi, Taiwan;; 2 Department of Emergency Medicine, Chang Gung Memorial Hospital, Chiayi, Taiwan;; 3 Department of Cardiology, Chang Gung Memorial Hospital, Chiayi, Taiwan;; 4 Department of Nursing, Chang Gung University of Science and Technology, Chiayi, Taiwan;; 5 Department of Laboratory Medicine, New Taipei Municipal Tu Cheng Hospital, Taipei, Taiwan;; 6 Department of Pulmonary and Critical Care Medicine, Chang Gung Memorial Hospital, Chiayi, Taiwan;; 7 Graduate Institute of Clinical Medical Sciences, College of Medicine, Chang Gung University, Taoyuan City, Taiwan;; 8 Graduate Institute of Gerontology and Health Care Management, Chang Gung University of Science and Technology, Taoyuan, Taiwan.

**Keywords:** dehydration, water intake, elderly, lung function impairment

## Abstract

**BACKGROUND:**

This study aimed to investigate the association between water intake and lung function impairment, focusing on the potential effect of dehydration on respiratory health in elderly adults.

**METHODS:**

A community-based, cross-sectional study was conducted in rural regions of Taiwan between March and December 2022. A total of 888 participants were enrolled. Data collection included lung function tests, physiological measurements, demographic characteristics, and medical histories. The effects of various variables on lung function were analysed across different age groups.

**RESULTS:**

Both restrictive and obstructive lung function impairment were significantly associated with older age, low water intake, and lower educational attainment. Restrictive lung function impairment was additionally correlated with the presence of cardiometabolic diseases, whereas obstructive lung function impairment was associated with male sex and smoking. Among participants aged over 65 years, low water consumption was associated with increased odds of restrictive and obstructive lung function impairment.

**CONCLUSION:**

Low water intake is associated with an increased risk of both restrictive and obstructive lung function impairment, particularly in elderly adults. Public health interventions promoting adequate hydration may help preserve pulmonary function in elderly population.

Lung function impairment represents a significant and growing public health concern, encompassing a wide range of respiratory conditions such as chronic obstructive pulmonary disease (COPD) and restrictive lung diseases. These pulmonary impairments are influenced by a complex interplay of genetic factors, environmental exposures, and lifestyle behaviours.^1–3^ Promoting respiratory health requires a comprehensive understanding of modifiable risks, particularly within ageing populations where the burden of chronic diseases is rapidly escalating.

Among lifestyle factors, hydration status has emerged as a crucial but often overlooked component influencing pulmonary outcomes. Proper hydration plays an essential role in maintaining effective mucociliary clearance, sustaining airway surface hydration, and preserving overall lung function.^4–6^ Dehydration is associated with impaired lung function in cystic fibrosis,^7^ and hydration status has been identified as a useful predictor of pulmonary function in patients with COPD.^6,8,9–11^ Systemic dehydration, whether due to exercise in hot environments or prolonged fluid restriction, negatively affects pulmonary function,^12^ whereas maintaining hydration may lower pneumonia-related mortality.^13^

Despite these findings, most existing literature focuses on obstructive lung disease. The relationship between hydration status and restrictive lung impairments, particularly among the elderly population, remains underexplored. Moreover, environmental and occupational factors, such as prolonged outdoor work and limited access to water sources, may exacerbate dehydration risks, especially in the rural elderly population engaged in agricultural activities.

In this community-based study, we aim to explore the association between water intake and lung function impairment among elderly adults in rural Taiwan. By examining the association between hydration habits and both obstructive and restrictive lung function impairments, we aim to seek new evidence that may contribute to strategies for promoting lung health among the ageing population and guiding future community health interventions.

## METHODS

This community-based, cross-sectional study was conducted in rural regions along Taiwan’s western coastline. The research was part of a community health promotion programme developed through a collaboration between a medical centre and local health authorities. Data were collected from March to December 2022. Study participants included individuals aged 40 years or older, who were currently engaged in small-scale farming, independently mobile, and able to provide informed consent. The exclusion criteria were an inability to answer questions or incomplete data.

Participants were invited to community activity centres for standardised health evaluations. Demographic data (age, sex, and education level) and medical histories, including self-reported diagnoses of chronic diseases such as hypertension, diabetes, hyperlipidaemia, coronary artery disease, and stroke, were collected using structured questionnaires. Cardiometabolic disease (CMD) was defined as the presence of at least two diagnoses of these conditions.^14^ Body measurements and physiological parameters were recorded and classified using the following cut-off points: waist circumference (WC) in males/females ≥ 90/80 cm; glycosylated haemoglobin (HbA1c) ≥ 6%; fasting low-high-density lipoprotein cholesterol (HDL-C) < 40/50 mg/dL for males/females; systolic/diastolic blood pressure (SBP/DBP) ≥ 130/85 mmHg; and fasting triglycerides (TGs) ≥150 mg/dL.^15^

Pulmonary function was assessed using a dry rolling-seal spirometer (Pony Fx-EN13485, COSMED), with calibration conducted daily via a precision syringe. Measurements were performed in accordance with the American Thoracic Society’s recommendations.^16^ Each participant completed five to six maximal forced expiratory manoeuvers, and three primary indices were recorded: forced vital capacity (FVC), the predicted percentage of FVC, and the forced expiratory volume in 1 s to FVC ratio (FEV1/FVC). Classification of lung function was as follows: normal (FEV1/FVC ≥ 70% and predicted FVC ≥ 80%), obstructive lung impairment (FEV1/FVC < 70% and predicted FVC ≥ 80%), and restrictive lung impairment (FEV1/FVC ≥ 70% with predicted FVC < 80%).^17^

Lifestyle factors were assessed by a standardised questionnaire. Information collected included smoking history (ever smoker, current/former smoker, never smoker), frequency of vegetable and fruit consumption (daily servings), water intake (daily volume), and engagement in regular physical activity (minimum 150 min per week or ≥30 min per session at least three times weekly). Responses regarding diet, hydration, and exercise behaviours were categorised into ‘lower frequency’ (rarely/occasionally) and ‘higher frequency’ (frequently/always) groups.

Multinomial logistic regression analysis was employed to investigate the association between health-related variables and categories of lung function impairment, with the normal lung function group serving as the reference. Interaction terms were included to assess whether associations differed by age groups. Significant predictors from univariate analyses were subsequently included in multivariable models. Data analyses were conducted using R software version 4.0.2 (The R Foundation for Statistical Computing, Vienna, Austria). Results are presented as odds ratios (ORs) with corresponding 95% confidence intervals (CIs), with *P* values < 0.05 considered statistically significant and *P* values < 0.1 deemed marginally significant.

### Ethical statement

Approval for this study was obtained from the Institutional Review Board of Chang Gung Memorial Hospital (IRB No: 202002186B0). Written informed consent was secured from all participants prior to data collection, ensuring compliance with ethical standards as outlined in the Declaration of Helsinki.

## RESULTS

A total of 888 rural adult residents were enrolled in this study. Lung function tests revealed that 355 participants (40%) had restrictive lung function impairment, whereas 94 individuals (10.6%) exhibited obstructive lung impairment. The remaining 439 participants (49.4%) had normal lung function. The mean age of the participants was 65.8 ± 14.55 years. Approximately 40.5% of the participants were male, and 59.7% were aged 65 years or older. Educational attainment was relatively limited, with 58.9% of participants having completed education beyond the elementary level. Lifestyle assessments showed that 17.2% of participants were classified as current or former smokers (Table 1). 58.7% of participants reported low-frequency consumption of fruits and vegetables, while 38.6% had inadequate daily water intake, and 55.5% had low levels of regular physical activity.

**TABLE 1.** Demographic characteristics and health-related behaviours based on the lung function test (*N* = 888).

Variables	Normal (*n* = 439)	Restrictive (*n* = 355)	Obstructive (*n* = 94)	*P* ^A^	*P* ^B^
Age				<0.01	<0.01
>65	223 (50.8)	227 (63.9)	80 (85.1)		
<65	216 (49.2)	128 (36.1)	14 (14.9)		
Sex				0.48	<0.01
Male	174 (39.6)	132 (37.2)	54 (57.4)		
Female	265 (60.4)	223 (62.8)	40 (42.6)		
Lower education^C^				<0.01	<0.01
Yes	213 (48.7)	233 (65.6)	77 (81.9)		
No	224 (51.3)	122 (34.4)	17 (18.1)		
Ever smoker (current/former)				0.86	0.01
Yes	71 (16.2)	56 (15.8)	26 (27.7)		
No	366 (83.8)	299 (84.2)	68 (72.3)		
Vegetable and fruit (5 servings/day)				0.3	0.17
Rarely	133 (30.4)	109 (30.7)	34 (36.2)		
Occasionally	112 (25.6)	107 (30.1)	26 (27.7)		
Frequently	57 (13)	43 (12.1)	10 (10.6)		
Always	135 (30.9)	96 (27)	24 (25.5)		
Water intake (>1,500 mL/day)				0.08	0.04
Rarely	79 (18.1)	68 (19.2)	20 (21.3)		
Occasionally	72 (16.5)	79 (22.3)	25 (26.6)		
Frequently	83 (19)	66 (18.6)	15 (16)		
Always	203 (46.5)	142 (40)	34 (36.2)		
Regular exercise				0.07	0.45
Rarely	186 (42.6)	128 (36.1)	33 (35.1)		
Occasionally	72 (16.5)	55 (15.5)	19 (20.2)		
Frequently	48 (11)	56 (15.8)	14 (14.9)		
Always	131 (30)	116 (32.7)	28 (29.8)		
WC (M > 90 cm, F > 80 cm)				0.08	0.42
Yes	270 (61.5)	240 (67.6)	62 (66)		
No	169 (38.5)	115 (32.4)	32 (34)		
HbA1c (>6%)				0.02	0.31
Yes	176 (40.2)	168 (47.3)	44 (46.8)		
No	262 (59.8)	187 (52.7)	50 (53.2)		
HDL-C (M < 40 mg/dL, F < 50 mg/dL)				0.47	0.13
Yes	112 (25.5)	104 (29.4)	20 (21.3)		
No	327 (74.5)	250 (70.6)	74 (78.7)		
SBP/DBP (>130/85 mmHg)				0.41	0.94
Yes	282 (64.2)	221 (61.4)	57 (64)		
No	157 (35.8)	139 (38.6)	32 (36)		
TG (>150 mg/dL)				0.27	0.73
Yes	123 (28)	112 (31.5)	28 (29.8)		
No	316 (72)	242 (68.4)	66 (70.2)		
CMDs (at least two diagnosis)				<0.01	0.1
Yes	55 (12.6)	81 (22.8)	18 (19.1)		
No	382 (87.4)	274 (77.2)	76 (80.9)		
FEV1		0.81 (0.79–0.84)	0.79 (0.76–0.81)	<0.01	<0.01
FVC		0.92 (0.80–1.07)	0.97 (0.85–1.12)	0.27	0.72
FEV1/FVC		1.05 (1.02–1.07)	0.06 (0.06–0.07)	<0.01	<0.01
Age × vegetable and fruit^D^		1.17 (1.04–1.31)^E^	1.52 (1.28–1.81)^F^	<0.01	<0.01
Age × water^G^		1.35 (1.18–1.55)^E^	1.64 (1.35–1.99)^F^	<0.01	<0.01
Age × exercise^H^		1.14 (1.02–1.28)^E^	1.43 (1.20–1.7)^F^	0.03	<0.01

WC = waist circumference; HbA1c = glycosylated haemoglobin; HDL-C = high-density lipoprotein cholesterol; SBP/DBP = systolic/diastolic blood pressure; TG = triglyceride; CMDs = cardiometabolic diseases; FEV1 = forced expiratory volume in 1 s; FVC = forced vital capacity; FEV1/FVC = forced expiratory volume in 1 s to FVC ratio.

A The *P* value for the variables in relation to the Restrictive group in univariate multinomial logistic regression analysis.

B The *P* value for the variables in relation to the Obstructive group in univariate multinomial logistic regression analysis.

C Below or equal to elementary school education.

D Over 65 years old with lower frequency of vegetable and fruit consumption.

E The OR (95%) for the interaction terms in relation to the Restrictive group in univariate multinomial logistic regression analysis.

F The OR (95%) for the interaction terms in relation to the Obstructive group in univariate multinomial logistic regression analysis.

G Over 65 years old with lower frequency of water intake.

H Over 65 years old with relatively low levels of physical activity.

Factors associated with restrictive lung function impairment included older age (>65 years), lower education (*P* < 0.01), low-frequency water intake (*P* < 0.1), low-frequency exercise (*P* < 0.1), larger waist circumference, elevated HbA1c levels, and the presence of CMD (*P* < 0.01). For obstructive lung function impairment, older age (*P* < 0.01), male sex (*P* < 0.01), lower educational level (*P* < 0.01), smoking history (*P* < 0.01), and low-frequency water intake (*P* < 0.05) were identified as significant risk factors (Table 1). Univariate analysis of the interaction with age revealed that being aged > 65 with a low frequency of vegetable and fruit consumption (*P* < 0.01) and a low frequency of water intake (*P* < 0.01) were associated with both obstructive and restrictive lung function impairment. A low frequency of regular exercise (*P* < 0.01) was associated with obstructive lung function impairment (Table 1).

**FIGURE 1.**
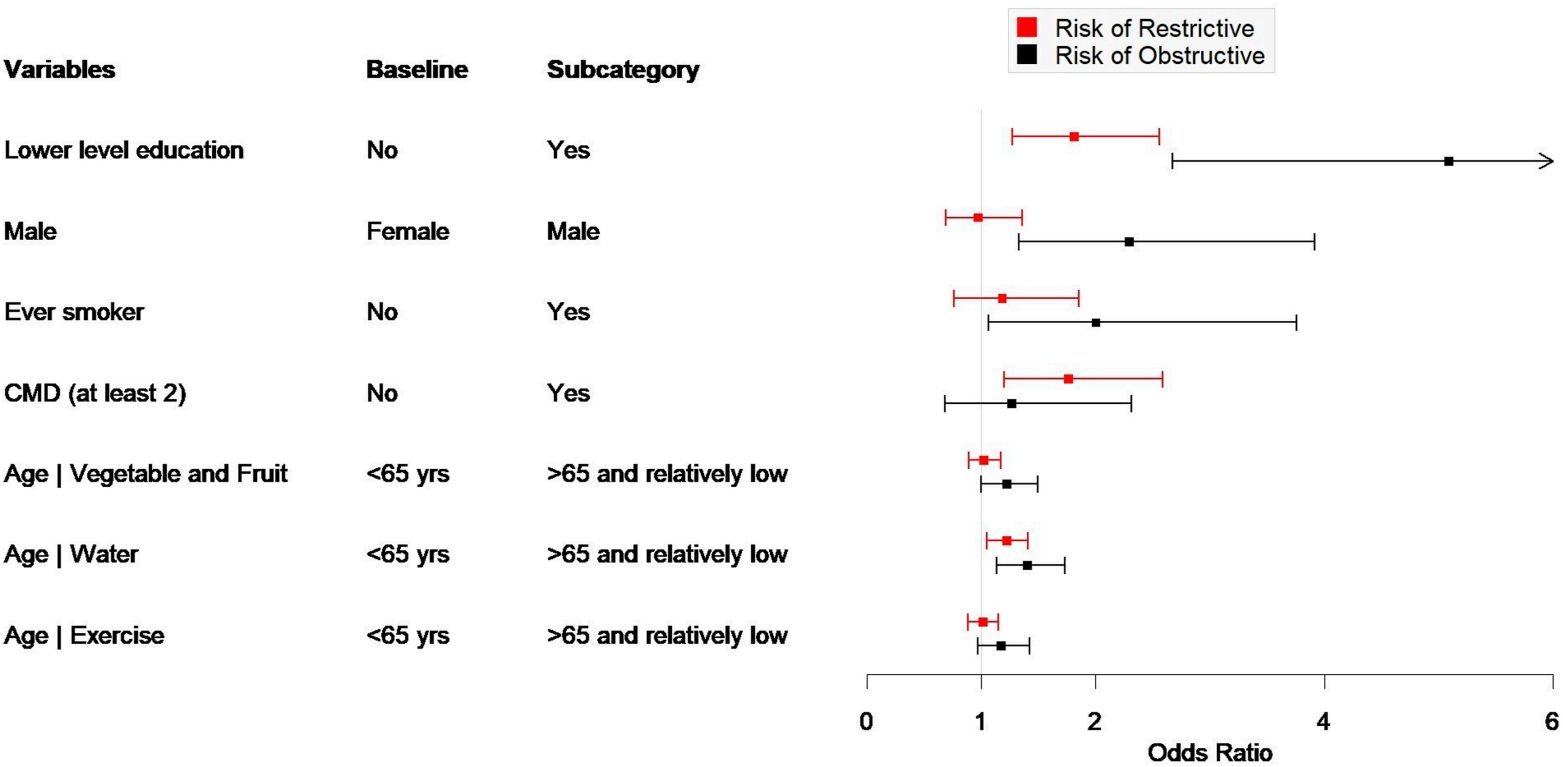
Factors associated with restrictive and obstructive type of lung impairment compared to normal lung function.

**FIGURE 2.**
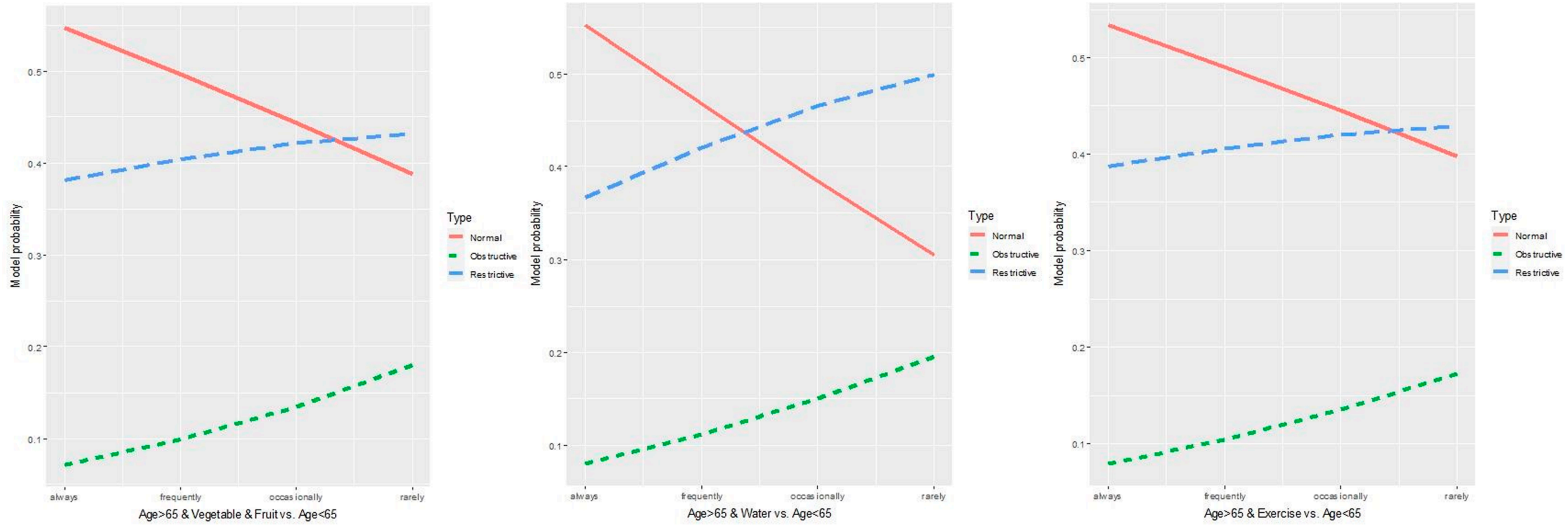
Insufficient vegetables/fruits, low water intake, and lack of exercise have adverse effects on the lung function test of the older adults.

Multinomial logistic regressions revealed that lower educational attainment (OR = 1.81, 95% CI: 1.27–2.56, *P* < 0.01) and the presence of CMD (OR = 1.76, 95% CI: 1.20–2.59, *P* < 0.01) were associated with an increased likelihood of restrictive lung function impairment. Among participants older than 65 years, low water intake was independently associated with restrictive lung impairment (OR = 1.22, 95% CI: 1.05–1.41, *P* < 0.01) (Table 2; Figures 1 and 2).

**TABLE 2.** Multinomial logistic regressions (reference group = normal).

	Model 1		Model 2		Model 3	
	Beta (SE)	OR (95% CI)	Beta (SE)	OR (95% CI)	Beta (SE)	OR (95% CI)
Restrictive type						
Education^A^	0.59 (0.18)	1.81 (1.27–2.56)**	0.47 (0.17)	1.59 (1.15–2.21)**	0.61 (0.17)	1.84 (1.31–2.58)**
Sex	−0.03 (0.17)	0.97 (0.69–1.36)	−0.02 (0.17)	0.98 (0.7–1.38)	−0.03 (0.17)	0.97 (0.69–1.37)
Ever smoker	0.17 (0.23)	1.18 (0.76–1.85)	0.18 (0.23)	1.19 (0.76–1.87)	0.17 (0.23)	1.18 (0.75–1.84)
HbA1c (>6%)	0.22 (0.15)	1.24 (0.92–1.67)	0.21 (0.15)	1.23 (0.91–1.66)	0.22 (0.15)	1.24 (0.92–1.67)
CMDs (at least 2)	0.57 (0.20)	1.76 (1.2–2.59)**	0.56 (0.20)	1.75 (1.19–2.57)**	0.57 (0.20)	1.77 (1.20–2.59)**
Interaction terms (reference: age < 65)						
Vegetable and fruit^B^	0.02 (0.07)	1.02 (0.89–1.17)				
Water^C^			0.2 (0.08)	1.22 (1.05–1.41)**		
Exercise^D^					0.007 (0.07)	1.01 (0.88–1.15)
Obstructive type						
Education^A^	1.62 (0.33)	5.09 (2.67–9.72)**	1.62 (0.32)	5.05 (2.72–9.38)**	1.71 (0.32)	5.55 (2.96–10.39)**
Sex	0.83 (0.27)	2.29 (1.33–3.92)**	0.89 (0.28)	2.43 (1.41–4.18)**	0.84 (0.27)	2.32 (1.36–3.97)**
Ever smoker	0.69 (0.32)	2 (1.06–3.76)*	0.67 (0.32)	1.95 (1.03–3.68)*	0.68 (0.32)	1.97 (1.05–3.7)*
HbA1c (>6%)	−0.03 (0.24)	0.97 (0.6–1.57)	−0.05 (0.25)	0.95 (0.59–1.54)	−0.03 (0.25)	0.97 (0.6–1.58)
CMD (at least 2)	0.23 (0.31)	1.26 (0.68–2.31)	0.17 (0.31)	1.19 (0.64–2.2)	0.19 (0.31)	1.21 (0.66–2.22)
Interaction terms (reference: age< 65)						
Vegetable and fruit^B^	0.20 (0.1)	1.22 (1–1.49)*				
Water intake^C^			0.33 (0.11)	1.4 (1.13–1.73)**		
Exercise^D^					0.16 (0.10)	1.17 (0.97–1.42)^+^

*P < 0.05.

**P < 0.01.

OR = odds ratio, CI = confidence interval; HbA1c = glycosylated haemoglobin; CMD = cardiometabolic disease.

A Below or equal to elementary school level.

B Over 65 years old with lower frequency of vegetable and fruit consumption.

C Over 65 years old with lower frequency of water intake.

D Over 65 years old with relatively low levels of physical activity.

Obstructive lung function impairment was associated with low educational attainment (OR = 5.09, 95% CI: 2.67–9.72, *P* < 0.01), male sex (OR = 2.29, 95% CI: 1.33–3.92, *P* < 0.01), and ever-smokers (OR = 2.0, 95% CI: 1.06–3.76, *P* < 0.05). Low water intake (OR = 1.4, 95% CI: 1.13–1.73, *P* < 0.01) and lower vegetable and fruit consumption (OR = 1.22, 95% CI: 1.00–1.49, *P* < 0.05) were associated with obstructive lung impairment in elderly participants (Table 2; Figures 1 and 2).

## DISCUSSION

This community-based study explored the association between daily water intake, lifestyle factors, and lung function impairment in the elderly population. The study participants resided in rural areas of Taiwan, where the majority of the local population are elderly individuals engaged in agriculture. Nearly half of the participants exhibited lung function abnormalities, with restrictive patterns being more common than obstructive ones. Among participants over 65 years old, the prevalence of pulmonary function impairment was even higher (57.9%), with restrictive-type impairment accounting for 42.8%. Age was found to be a factor significantly associated with both obstructive and restrictive lung impairments in our study. Ageing has been linked to alterations in the structural and mechanical properties of the lungs, including reductions in alveolar surface area, elastic recoil, respiratory muscle strength, and chest wall compliance, contributing to declines both in FEV1 and FVC.^8,9^ The vascular stiffening and diminished oxygen exchange associated with ageing further exacerbate respiratory inefficiency.^8–11,18^ Among our participants, 85.1% with obstructive lung function impairment and 63.9% with restrictive impairment were over 65 years old, highlighting a strong association between advanced age and pulmonary function decline. Factors related to old age and impaired lung function are important for the preservation of pulmonary function, especially in an ageing society.

Low water intake was significantly associated with both obstructive and restrictive lung impairment, particularly among the elderly adults in this study. Dehydration has been linked to reduced mucociliary clearance, impaired epithelial barrier integrity, and increased airway resistance due to thicker mucus and impaired secretion dynamics.^19–23^ Systemic dehydration may further impair pulmonary function by reducing lung volumes and increasing residual volumes (RVs).^19,20,24^ Individuals with pre-existing respiratory diseases may exhibit stronger associations between hydration status and pulmonary function. In contrast, adequate hydration is important for preserving mucus clearance and ensuring airway patency. Declines in pulmonary function parameters such as FVC, functional residual capacity, and RV have been rapidly reversed through water supplementation in healthy individuals.^19^ Maintaining adequate hydration may be a potential strategy for preserving lung function in elderly adults. However, for individuals engaged in manual labour, it is difficult to quantify daily water intake or determine whether their current fluid consumption is sufficient. Access to drinking water may be inconsistent in the workplace, which could result in prolonged periods of dehydration before adequate fluid replenishment is achieved. Recall bias and measurement errors in estimating water intake may also exist when using self-reported questionnaires. Therefore, further longitudinal studies are warranted to clarify the causal relationship between hydration status and pulmonary function impairment.

Other factors contributing to pulmonary function impairment included lower educational attainment, CMDs, and unhealthy lifestyle behaviours including smoking and insufficient exercise. Lower educational attainment was related to both restrictive and obstructive lung impairment. Lower educational attainment may be associated with reduced health literacy, lower participation in preventive health behaviours, and higher prevalence of modifiable risk factors including smoking and sedentary lifestyle.^25,26^ Limited educational attainment may necessitate delivering health education over longer periods and through repeated sessions to achieve the desired outcomes, while elderly individuals in rural areas may face relatively greater difficulty in accessing health care information. CMDs were significantly associated with restrictive lung impairment in our cohort, supporting existing literature indicating that metabolic and cardiovascular disorders impair pulmonary structure and function.^27,28^ Medications commonly used in the management of CMDs, including diuretics, may lead to dehydration. In addition, suboptimal management and complications of CMDs may further contribute to fluid imbalance, manifesting as hyperosmolar hyperglycaemic state or dehydration in patients with post-stroke sequelae. Accurate assessment and continuous monitoring of fluid status, however, remain particularly challenging in elderly populations.

Although limited studies have investigated the relationship between lifestyle factors and lung function impairment, our findings suggest that participants over 65 years old with low physical activity levels and infrequent fruit and vegetable consumption had a higher risk of lung dysfunction. Previous research has shown that healthy dietary patterns help preserve lung function in smokers without respiratory disease, whereas unhealthy diets are linked to pulmonary decline.^29^ Furthermore, interventions combining high-intensity interval training and resistance training have been shown to improve systemic oxidative stress and muscle function in older adults with COPD.^30^ These findings highlight the association between proper nutrition, regular exercise, and pulmonary health.

Several limitations should be considered. First, the cross-sectional design of the study limits the ability to establish causal relationships; therefore, the associations between hydration, lifestyle factors, and pulmonary function should not be interpreted as causal. Second, our study population consisted primarily of elderly farmers in rural areas, which may limit generalisability to urban or non-agricultural populations. Caution is warranted when applying these findings beyond similar rural settings. Third, water intake and lifestyle behaviours were assessed by self-reported questionnaires, which may be subject to measurement error and recall bias. Such inaccuracies could attenuate or exaggerate observed associations, potentially influencing the strength of the reported relationships. Finally, although we adjusted for several confounders, unmeasured variables such as environmental exposures, comorbidities, and medication use affecting hydration status may have influenced the results and affected the validity of our findings.

## CONCLUSION

Our findings indicate that low water intake is associated with an increased risk of both restrictive and obstructive lung function impairment, particularly in elderly adults. Ageing, low educational attainment, CMDs, and unhealthy lifestyle habits also contribute significantly to impaired lung function. Public health interventions promoting adequate hydration, healthy dietary habits, and physical activity may help preserve pulmonary function and mitigate respiratory morbidity in elderly populations. Future longitudinal studies with diverse populations and objective biomarkers are warranted to clarify the causal effects of hydration status on pulmonary health outcomes.
